# Thymoma without Myasthenia Gravis Showing Size Fluctuation in Parallel to Alopecia Areata Activity: A Case Report

**DOI:** 10.5761/atcs.cr.25-00082

**Published:** 2025-07-15

**Authors:** Keisuke Fujimoto, Koichiro Kenzaki, Takako Kubo, Toru Sawada, Shoko Norimura, Kazumasa Miura, Akiyoshi Yamamoto

**Affiliations:** 1 Chest Medical Center, Takamatsu Red Cross Hospital, Takamatsu, Kagawa, Japan

**Keywords:** alopecia areata, thymoma, myasthenia gravis, surgery, autoimmune disease

## Abstract

Thymomas are commonly associated with autoimmune diseases such as myasthenia gravis (MG), pure red cell aplasia, and hypogammaglobulinemia, while those associated solely with alopecia areata (AA) are extremely rare. A 55-year-old woman with AA underwent chest computed tomography, which revealed a 33-mm anterior mediastinal cystic mass with fluctuating size. She was referred to our department for evaluation of a suspected cystic thymoma. The patient underwent thoracoscopic tumor resection under general anesthesia with isolated lung ventilation in the left lateral decubitus position. The operation lasted 81 minutes with minimal blood loss, and her postoperative course was uneventful. Histopathology confirmed a type B2-dominant thymoma. Notably, the patient’s AA improved rapidly after surgery and did not recur for at least 3 years. This case strongly suggests a potential immunological relationship between AA and thymoma, though further research is needed to confirm this relationship.

## Introduction

Alopecia areata (AA) is an acquired disease that results in hair loss in a circular pattern and affects approximately 1.7% of the population at least once in their lifetime.^1)^ It is thought to trigger an autoimmune reaction targeting autoantigens derived from hair follicles, and the disease is often associated with autoimmune diseases. In severe cases, alopecia patches often expand with cycles of exacerbations and remissions.^2)^ Thymomas are also associated with autoimmune diseases, including myasthenia gravis (MG), pure red cell aplasia, and hypogammaglobulinemia. Although AA is reported to occur in 0.5%–17% of patients,^1)^ there are few reports of thymoma associated with AA without concomitant MG. We report herein a rare case of AA that improved within approximately 3 weeks after thymoma resection and has not recurred for 3 years.

## Case Report

A 55-year-old woman presented to the dermatology department 3 years before this event with complaints of progressive hair loss that was unresponsive to topical treatments. A punch biopsy of the parietal scalp confirmed a diagnosis of AA. As there was no improvement with squaric acid dibutylester sensitization therapy, intralesional triamcinolone injections (40 mg) were administered for 6 months, resulting in clinical improvement, after which follow-up was discontinued.

However, 1 year later, the patient returned with rapidly worsening AA, requiring resumption of intralesional triamcinolone injections (20 mg). During routine computed tomography (CT) of the chest for breast cancer surveillance, an enlarged anterior mediastinal mass was incidentally detected. The patient was subsequently referred to our department for further evaluation and treatment. The mediastinal mass had already been identified at the previous hospital; however, since it demonstrated temporary size reduction, the patient was followed up conservatively without active therapeutic intervention. Her medical history included left breast cancer (treated with mastectomy and reconstruction), endometriosis, and colonic polyp. She reported smoking 35 cigarettes per day for 10 years, no alcohol consumption, and was taking exemestane.

**Fig. 1:**
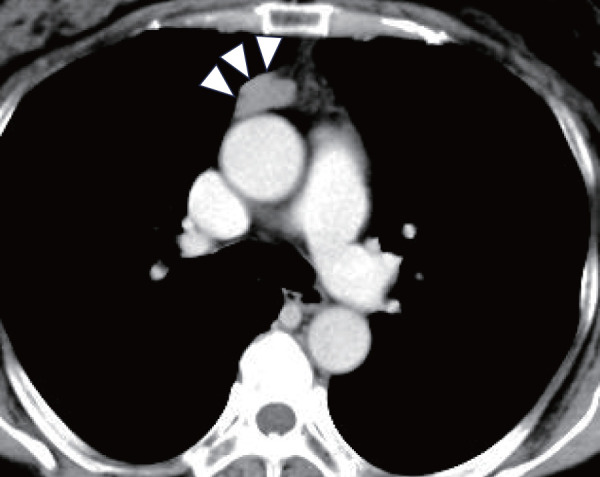
Contrast-enhanced chest CT demonstrating a lobulated cystic mass (23 × 14 × 33 mm) in the anterior mediastinum, broadly contacting the pericardium. The lesion exhibits homogeneous internal density with minimal contrast enhancement, raising suspicion for a thymic or pericardial cyst (arrows). CT: computed tomography

**Fig. 2:**
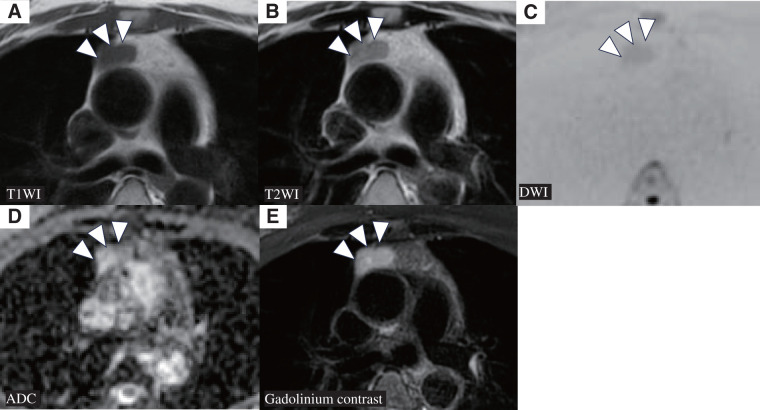
Contrast-enhanced chest MRI findings. (**A**) T1-weighted imaging showing uniform low signal intensity. (**B**) T2WI demonstrating pale, homogeneous high signal intensity without fat suppression. (**C**, **D**) DWI presenting pale high signal intensity without ADC reduction. (**E**) Gadolinium-enhanced MRI exhibiting mild enhancement limited to the capsule-like peripheral structure, with no internal enhancing component. ADC: apparent diffusion coefficient; DWI: diffusion-weighted imaging; MRI: magnetic resonance imaging; T2WI: T2-weighted imaging

Laboratory tests revealed that anti-acetylcholine receptor antibody (AChR-Ab) was weakly positive at 0.5 nmol/L (normal <0.3). Nerve conduction studies showed no significant waning. Contrast-enhanced chest CT revealed a 23 × 14 × 33-mm cystic mass in the anterior mediastinum, broadly adjacent to the pericardium with lobulated margins, homogeneous internal density, and minimal contrast enhancement, suggestive of a thymic or pericardial cyst (**Fig. 1**). Contrast-enhanced chest magnetic resonance imaging showed uniform low T1 signal (**Fig. 2A**), homogeneous high T2 signal without fat suppression (**Fig. 2B**), diffusion restriction without apparent diffusion coefficient reduction (**Figs. 2C** and **2D**), and mild peripheral enhancement without internal enhancement (**Fig. 2E**).

Although the AChR-Ab was weakly positive, the patient had no symptoms such as ptosis and, therefore, did not meet the diagnostic criteria for MG. Based on the imaging findings, the lesion was suspected to be a cystic mass in the anterior mediastinum without a solid component, possibly a thymic or pericardial cyst. However, given the possibility of a cystic thymoma, surgical resection was planned for both diagnostic and therapeutic purposes.

**Fig. 3:**
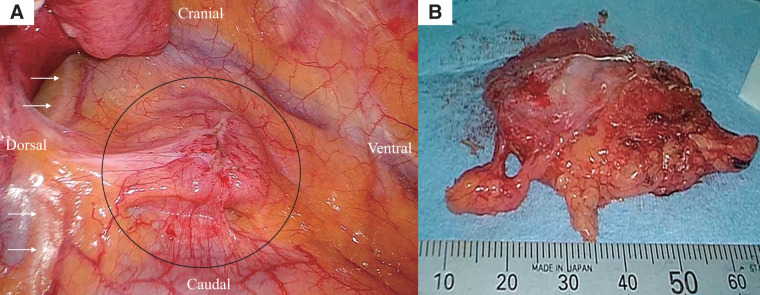
Surgical findings. (**A**) The tumor was located ventral to the phrenic nerve, showing mild adhesions. (**B**) Thoracoscopic resection of the tumor along with surrounding thymic and fatty tissues was performed due to suspicion of thymoma.

Surgery was performed under general anesthesia with isolated lung ventilation, in the left lateral decubitus position using a right thoracoscopic 3-port approach. The tumor was located ventral to the phrenic nerve (**Fig. 3A**), with mild adhesions, and given the potential for thymoma, the mass and surrounding thymic and fatty tissues were completely resected (**Fig. 3B**). The operation lasted 81 minutes with minimal blood loss.

**Fig. 4:**
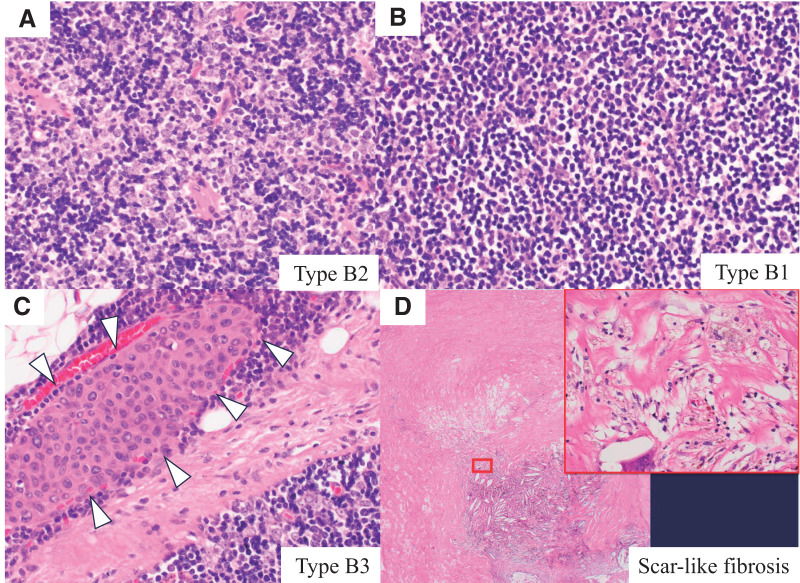
Histopathological findings of the resected thymoma. (**A**) Predominantly type B2 thymoma, characterized by epithelial cells in a lymphocyte-rich background. (**B**) Areas consistent with type B1 thymoma. (**C**) A small epithelial cluster (approximately 0.5 mm) resembling type B3 thymoma. (**D**) A fibrotic, scar-like area approximately 1.2 × 1.0 cm.

Histopathology revealed a lobulated tumor encapsulated in fibrous tissue. Histologically, the lesion was predominantly a type B2 thymoma, consisting of epithelial cells against a lymphocytic background (**Fig. 4**); however, areas consistent with type B1 thymoma were also observed (**Fig. 4B**), as was a small epithelial cluster (approximately 0.5 mm) resembling type B3 (**Fig. 4C**). A scar-like fibrotic area measuring approximately 1.2 × 1.0 cm was also noted (**Fig. 4D**). The final diagnosis was thymoma type B2 > B1. According to the Union for International Cancer Control 8th edition TNM classification, the staging was pT1a, pNX, cM0; Masaoka classification—stage II; Masaoka–Koga classification—stage IIb.

The thoracic drain was removed on postoperative day 1, and the patient was discharged on postoperative day 4. The symptoms of AA showed marked improvement within 3 weeks after surgery and did not recur during the subsequent 3 years. The thymoma remained recurrence-free, and the patient continued to undergo regular follow-up at our outpatient clinic.

## Discussion

AA is an autoimmune disease characterized by hair loss resulting from autoimmune-mediated damage to the hair follicles. It frequently occurs concomitantly with other autoimmune diseases, such as MG, lupus erythematosus, and vitiligo.^1,2)^ Thymoma is also associated with autoimmune diseases, including MG, pure red cell aplasia, and hypogammaglobulinemia, and occurs in approximately one-third of patients with thymoma.^3)^ AA is reported to occur concomitantly with thymoma in 0.5%–17% of cases; however, most instances also involve MG. Cases of thymomas associated exclusively with AA are extremely rare, and the present case represents only the third reported instance.^1,4)^

**Fig. 5:**
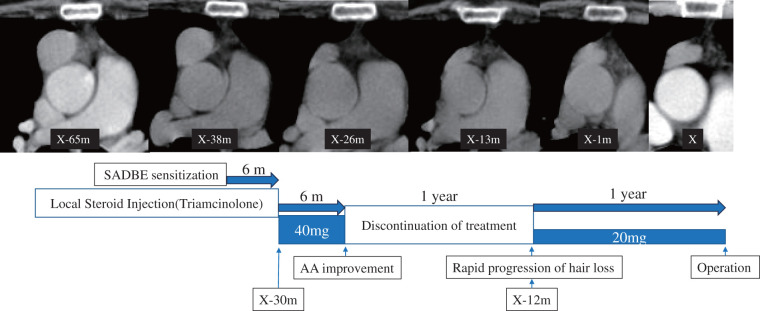
Patient’s clinical course demonstrating the synchronization between AA severity and thymoma size. Intralesional triamcinolone injections led to AA improvement and coincided with thymoma shrinkage. The tumor size remained unchanged during periods of AA stability without treatment. However, rapid tumor enlargement occurred simultaneously with AA exacerbation. Surgical thymoma resection resulted in dramatic AA improvement within approximately 3 weeks, with no recurrence during 3 years of postoperative follow-up. AA: alopecia areata; m: months; SADBE: squaric acid dibutylester; X: referral to our department

In our patient, fluctuations in the size of the anterior mediastinal mass closely mirrored changes in AA severity, a phenomenon that has not been previously reported. Intralesional triamcinolone injections improved AA while simultaneously reducing tumor size. Furthermore, during the period when AA was stable without treatment, the tumor size remained unchanged. However, a rapid increase in tumor size occurred when the AA was exacerbated. Following thymoma resection, AA dramatically improved within approximately 3 weeks, with no recurrence observed in the subsequent 3-year follow-up period (**Fig. 5**). The observed associations in the patient’s clinical course strongly suggest an immunological relationship between AA and thymoma, rather than a coincidental occurrence.

Spontaneous regression of thymomas has typically been reported in larger tumors (median diameter, 70 mm), presenting with symptoms such as chest pain or fever and showing adhesions to surrounding tissues and histopathological features of fibrosis or necrosis.^5–7)^ Although our patient’s thymoma was relatively small (maximum diameter 33 mm), it exhibited adhesions and fibrotic histological changes consistent with these features. Nevertheless, spontaneous regression alone would inadequately explain the precise synchronization observed between tumor size and AA severity, further supporting an immunological mechanism.

In our department, extended thymectomy is typically performed in cases of thymoma with positive AChR-Ab. However, in the present case, the lesion initially demonstrated size reduction on imaging, suggesting a thymic cyst as the most likely provisional diagnosis. Although thymoma was included in the differential diagnosis, the imaging findings and weakly positive AChR-Ab supported our decision to perform thymomectomy rather than an extended thymectomy. Postoperatively, the patient has not developed MG, and the antibody level has normalized.

Postoperative MG (PTMG), characterized by the onset of MG symptoms after thymectomy without preoperative manifestations, is uncommon. However, careful monitoring for this condition is recommended. Known risk factors for PTMG include preoperative AChR-Ab positivity, histological thymoma subtypes B1/B2/B3, and incomplete surgical resection.^8–10)^ Given the weak positivity for AChR-Ab and the histological subtypes (B1/B2/B3), careful long-term follow-up is warranted to monitor for the potential development of MG symptoms.^11–13)^

## Conclusions

Thymoma cases associated exclusively with AA, without concomitant MG, are extremely rare. To our knowledge, this is the first reported case where a thymoma associated solely with AA exhibited tumor size fluctuations correlating with AA severity. Given the rapid improvement in AA symptoms after thymoma resection, a potential immunological association between both conditions is strongly suggested. Further case studies and immunological research are needed, however, to confirm this relationship. High-risk patients with positive AChR-Ab and histological subtypes B1/B2/B3 require careful postoperative follow-up for potential development of PTMG.

## Declarations

### Ethics approval and consent to participate

Written informed consent was obtained from the patient to participate in this case report.

### Consent for publication

Written informed consent was obtained from the patient for the publication of this case report and accompanying images.

### Funding

Not applicable.

### Data availability

The data supporting the findings of this report are available from the corresponding author upon reasonable request.

### Authors’ contributions

KF: primary surgeon of the surgery and corresponding author. KK: primary assistant of the surgery, supervisor, and writer. TK, TS, SN, KM, and AY: assistants of the surgery and writers. All authors have read and agreed to the published version of the manuscript.

### Disclosure statement

The authors declare that they have no competing interests.
